# Exposure to a firefighting overhaul environment without respiratory protection increases immune dysregulation and lung disease risk

**DOI:** 10.1371/journal.pone.0201830

**Published:** 2018-08-21

**Authors:** Stephen J. Gainey, Gavin P. Horn, Albert E. Towers, Maci L. Oelschlager, Vincent L. Tir, Jenny Drnevich, Kenneth W. Fent, Stephen Kerber, Denise L. Smith, Gregory G. Freund

**Affiliations:** 1 Department of Animal Sciences, University of Illinois, Urbana, Illinois, United States of America; 2 Illinois Fire Service Institute, Champaign, Illinois, United States of America; 3 Division of Nutritional Sciences, University of Illinois, Urbana, Illinois, United States of America; 4 Department of Pathology, Program in Integrative Immunology and Behavior, University of Illinois College of Medicine, Urbana, Illinois, United States of America; 5 Roy J. Carver Biotechnology Center, University of Illinois, Urbana, Illinois, United States of America; 6 Division of Surveillance, Hazard Evaluations, and Field Studies, National Institute for Occupational Safety and Health, Cincinnati, Ohio, United States of America; 7 Director, UL Firefighter Safety Research Institute, Columbia, Maryland, United States of America; 8 Department of Health and Human Physiological Sciences, Skidmore College, Saratoga Spring, New York, United States of America; Auburn University College of Veterinary Medicine, UNITED STATES

## Abstract

Firefighting activities appear to increase the risk of acute and chronic lung disease, including malignancy. While self-contained breathing apparatuses (SCBA) mitigate exposures to inhalable asphyxiates and carcinogens, firefighters frequently remove SCBA during overhaul when the firegrounds appear clear of visible smoke. Using a mouse model of overhaul without airway protection, the impact of fireground environment exposure on lung gene expression was assessed to identify transcripts potentially critical to firefighter-related chronic pulmonary illnesses. Lung tissue was collected 2 hrs post-overhaul and evaluated via whole genome transcriptomics by RNA-seq. Although gas metering showed that the fireground overhaul levels of carbon monoxide (CO), carbon dioxide (CO_2_), hydrogen cyanine (HCN), hydrogen sulfide (H_2_S) and oxygen (O_2_) were within NIOSH ceiling recommendations, 3852 lung genes were differentially expressed when mice exposed to overhaul were compared to mice on the fireground but outside the overhaul environment. Importantly, overhaul exposure was associated with an up/down-regulation of 86 genes with a fold change of 1.5 or greater (p<0.5) including the immunomodulatory-linked genes S100a8 and Tnfsf9 (downregulation) and the cancer-linked genes, Capn11 and Rorc (upregulation). Taken together these findings indicate that, without respiratory protection, exposure to the fireground overhaul environment is associated with transcriptional changes impacting proteins potentially related to inflammation-associated lung disease and cancer.

## Introduction

Even as personal protective equipment (PPE) improves [1], the incidence and mortality from cancer in firefighters increases and is a leading cause of death [2]. Epidemiological evidence shows that firefighters have a greater risk of cancer when compared to the general population [2,3]. Firefighters in the United States respond to 1.2–1.4 million fires each year including approximately 475,000–500,000 structure fires [4]. Exposure to toxicants is possible during live fire responses, which can result in biological absorption of polycyclic aromatic hydrocarbons (PAHs) and benzene [5–7] and inhalation of carbon monoxide [8] and hydrogen cyanide [9]. Interestingly, in 2010, the International Agency for Research on Cancer (IARC) classified occupational exposure during firefighting as possibly carcinogenic to humans [10]. Part of the rational for this classification results from the lack of genotoxicity studies in animals that involves exposure to smoke from the combustion of structural materials. Even with substantial upgrades to PPE, such as SCBAs and turnout gear technology, firefighters are imperiled if SCBAs are compromised, not worn or removed [5,11–13]. While exposure risk is minimized with PPE [14,15], PPE usage is not universal for all phases of a response.

Currently, the highest risk of toxicant exposure appears to be during overhaul, since initial fire suppression is usually associated with heavy smoke and the obvious need for SCBA [16]. During overhaul, time spent searching for unextinguished fire inside structures can exceed 30 minutes and is most often coupled to improper or little use of respiratory protection [11,16]. Unfortunately, failure to use PPE during overhaul can result in contact with concealed carcinogens (like asbestos) due to fire- or firefighting-dependent structural damage [16]. In addition, smoke and/or fume inhalation is most prevalent during this period due to frequent abandonment of SCBA [17,18]. While the U.S. Fire Service has gained traction in limiting removal of SCBA, firefighters still make their own determination on when to utilize it based on heat stress, comfort or visual indications of clear air. Therefore, the purpose of this study was to examine the impact of unprotected respiratory exposure to the fireground during overhaul on mouse lung gene expression. It should provide insight to potential pathways linked to lung cancer development.

## Materials and methods

### Animals

The use of animals (S1 Checklist) was in accordance with the recommendation in the Guide for the Care and Use of Laboratory Animals of the National Institutes of Health and an Institutional Animal Care and Use Committee (IACUC) approved protocol (Protocol #15099) at the University of Illinois. C57BL/6J male mice (10 weeks old) were purchased from Jackson Laboratories (Bar Harbor, ME). Mice were group-housed (4 per cage) in shoebox cages (length 29.9 cm; width 18.4 cm; height 12.5 cm) and allowed free access to food and water, unless otherwise noted. Housing temperature (22 °C) and humidity (45–55%) were controlled as was a 12/12 h reversed dark-light cycle (light = 1000–2200 h). Animals were euthanized for tissue collection using CO_2_. Total number of mice used was 54.

### Live-fire scenario setup

Firefighting activities were conducted in a purpose-built live-fire research test structure. The structure, based on a design by a residential architectural company, was representative of a home constructed in the mid-twentieth century with walls and doorways separating all rooms and 2.4m ceilings. The structure had an approximate floor area of 111 m^2^ with 8 total rooms. Interior finish in the burn rooms was protected by gypsum board on the ceiling and walls. Furnishings were acquired from a lone source to afford inter-scenario standardization. The bedrooms, where the fires were ignited, were appointed with a double bed (covered with a foam mattress topper, comforter and pillow), stuffed chair, side table, lamp, dresser and flat screen television. Floors were covered with polyurethane foam padding and polyester carpet. Fires were ignited using the stuffed bedroom chair via remote ignition comprised of a match book electrically energized by fine wire heating. Each resultant flaming fire could grow until it approached early ventilation-limitation. Based on national averages, fire department dispatch was between 4–5 min after ignition for all scenarios. The structure was repaired/rebuilt after each scenario.

### Firefighting and overhaul

A team of 12 firefighters battled the fires involving two fully involved bedrooms. As soon as the fire was suppressed, and interior operations were completed (two simulated trapped occupants removed), mice were transported into the burned structure as overhaul operations by firefighters were initiated.

### Mouse groups/transport/housing

For each cohort (n = 18 mice/cohort), mice were placed into three groups (n = 6 each): 1) control (C) group, which never left the animal housing facility; 2) fireground (FG) group, which was taken to the fireground but placed in a portion of the structure that was uninvolved with the fire and overhaul activities: and 3) overhaul (OH) group, which was taken to the fireground and placed in the interior of the structure during overhaul (as described above). Three cohorts of mice were used, one for each of the three experiments performed on three separate days at approximately the same time of day (0800–0900). The FG and OH mouse groups were transported to the fireground, arriving 30 min prior to firefighting and were placed on a table approximately 25 m from the structure while active fire was being fought by the firefighters. Mice were housed in shoebox cages wrapped in heat-resistant AB Technology Group Knitted Fiberglass Plain Tape (Ogdensburg, NY) and 3M Silver Foil Tape 3340 (Maplewood, Minnesota), on 3.5 sides. Interior cage temperature was recorded using a Fisher Scientific (Hampton, NH) digital probe thermometer, and animals were visually monitored every 5 min throughout the exposure period for signs of pain or distress. Mouse groups were returned to the animal care facility 15 minutes after the conclusion of overhaul.

### Atmospheric data collection

Air concentrations of carbon monoxide (CO), carbon dioxide (CO_2_), hydrogen cyanide (HCN), hydrogen sulfide (H_2_S), and oxygen (O_2_) gases were quantified with a MX6 iBrid (Industrial Scientific; Pittsburg, PA) portable personal gas monitor. The meter was placed on top of the mouse cages in one of the fire rooms being overhauled by firefighters.

### RNA extraction and fragment analysis

Mouse lungs were harvested 2 hrs after the OH group was removed from the overhaul environment and immediately placed in Qiagen RNA*later* (Valencia, CA). RNA was extracted using the Qiagen miRNeasy Mini Kit including DNAase. RNA integrity was determined using an Applied Biosystems Fragment analyzer (Foster City, CA); all 54 samples had RQN score >7 and were defined as acceptable.

### Illumina RNA sequencing

RNAseq libraries were prepared with Illumina TruSeq Stranded RNA Sample Prep Kit (San Diego, CA) resulting in 5’ to 3’ strand-specific libraries. A single library was prepared from each sample. All libraries were then quantitated by qPCR and sequenced on seven lanes for 101 cycles using an Illumina HiSeq2500 100nt single-end read with the TruSeq SBS sequencing v3 kit. Fastq files were processed and demultiplexed with bcltofastq 1.8.4.

### RNAseq data and statistical analysis

Raw reads were checked for quality using FASTQC (v 0.11.2) then trimmed and filtered using Trimmomatic (v 0.33) to remove residual adapter content and low-quality bases (Phred quality score < 28). Trimmed/filtered reads were aligned to NCBI’s *Mus musculus* GRCm38.p3 genome and gene model annotation release 105 using STAR (v 2.4.2a). Post-alignment gene counts were then determined using featureCounts (v 1.4.3-pl) with multi-mapping reads excluded.

The gene-level read counts were then imported into R (v. 3.4.3) for statistical analyses. TMM normalization (Robinson and Oshlack 2010) in the edgeR package (Robinson et al. 2010; v 3.20.6) was used to normalize the counts to log2-transformed counts per million (logCPM), using the cpm function with prior count = 3. 25,525 genes without logCPM > log2 (1) in at least 5 samples were filtered out, leaving 16,261 genes to be analyzed for differential expression. TMM-values were re-calculated as well as logCPM normalized values with prior.count = 3 to use in down-stream analyses and visualizations.

Clustering of samples to check for outliers and batch effects was done using Principle Components Analysis [19]. We then performed surrogate variables analysis (sva) [20,21] using the sva package (v 3.26.0) [22],) to detect and remove artifacts like batch effects by creating eight surrogate variables (sv). The sv were added to the statistical model for the 3 treatment groups and differential expression testing [23] using the limma package’s (v 3.34.5) [24] “trend” approach because the variation in library sizes was less than the recommended 3-fold maximum [25]. A one-way ANOVA across the 3 groups was calculated, along with all three pairwise comparisons. Multiple hypothesis testing adjustment was done separately for each test using the False Discovery Rate (FDR) method [26]. While the sva method was judged to be the best way to correct the overall FG vs OH comparison for individual fire and other partially confounded batch effects, it does not allow us to pull individual FG vs OH comparisons for each fire. Therefore, we also made a separate statistical analysis for the 9-different treatments X fire groups + seven estimated surrogate variables and pulled out pairwise FG vs OH comparisons within each fire. Because we were mainly interested in comparing the numbers of genes differentially expressed between fires, we performed a global FDR correction across the three comparisons to ensure that a gene with the same raw p-value in different fires ended up with the same FDR p-value.

Functional annotation was taken from Bioconductor’s [27] org.Mm.eg.db package (v 3.5.0) using the respective Entrez Gene ID from NCBI. KEGG pathways were downloaded directly from http://www.kegg.jp/ using the KEGGREST package (v 1.18.0). Over-representation testing was done on KEGG pathways for specified gene sets using the GOstats (v 2.44.0) [28] and Category (v 2.44.0) packages. Statistical significance was assumed at FDR p < 0.05 unless otherwise noted.

## Results

### Overhaul environmental conditions

Table 1 shows that mouse cage temperature averaged 31.6 °C with differences between test fires ranging from 28.3–33.9 °C. Peak temperatures ranged from approximately 30.6–40.6 °C and occurred as the mice cages were introduced into the structure. Peak concentrations for CO_2_, HCN, H_2_S, and minimum level of O_2_ did not exceed the 10-hour NIOSH TWA levels. Peak CO did exceed NIOSH STEL and OSHA PEL TWA levels and remained under the NIOSH ceiling recommendation. Qualitatively, the overhaul environment appeared visually clear during each overhaul in contrast to dense smoke during the fire itself (data not shown).

**Table 1:** Environmental measurements for OH group mice during overhaul respective of mouse cohort/fire.

		Fire 1	Fire 2	Fire 3
**Exposure Time (min)**		18	15	15
**Temperature (°C)**	Average	33.9	28.3	32.8
	Peak	40.6	30.6	40.0
**CO (ppm)**	Average	26	28	37
	Peak	70	98	91
**CO_2_ (%)**	Average	0.02	0.02	0.01
	Peak	0.08	0.05	0.05
**HCN (ppm)**	Average	0.1	0.6	0.4
	Peak	1.1	2.8	1.7
**H_2_S (ppm)**	Average	0.0	0.1	0.5
	Peak	2.2	1.0	2.7
**O_2_ (%)**	Average	20.8	20.9	20.8
	Minimum	20.6	20.8	20.5

CO—NIOSH TWA 35ppm; OSHA TWA 50ppm; NIOSH C 200 ppm; IDLH 1200ppm

CO_2_—NIOSH TWA 0.5%; OSHA TWA 0.5%; NIOSH ST 3%; IDLH 4%

HCN—NIOSH ST 4.7 ppm OHSA TWA 10 ppm, IDLH 50 ppm

H_2_S—NIOSH C 10 ppm, OSHA C 20 ppm; IDLH 100 ppm

O_2_—typically alarm levels are set at <19.5%

### Principal component analysis (PCA) demonstrate distinct separations between C, FG and OH groups

**Fig 1:**
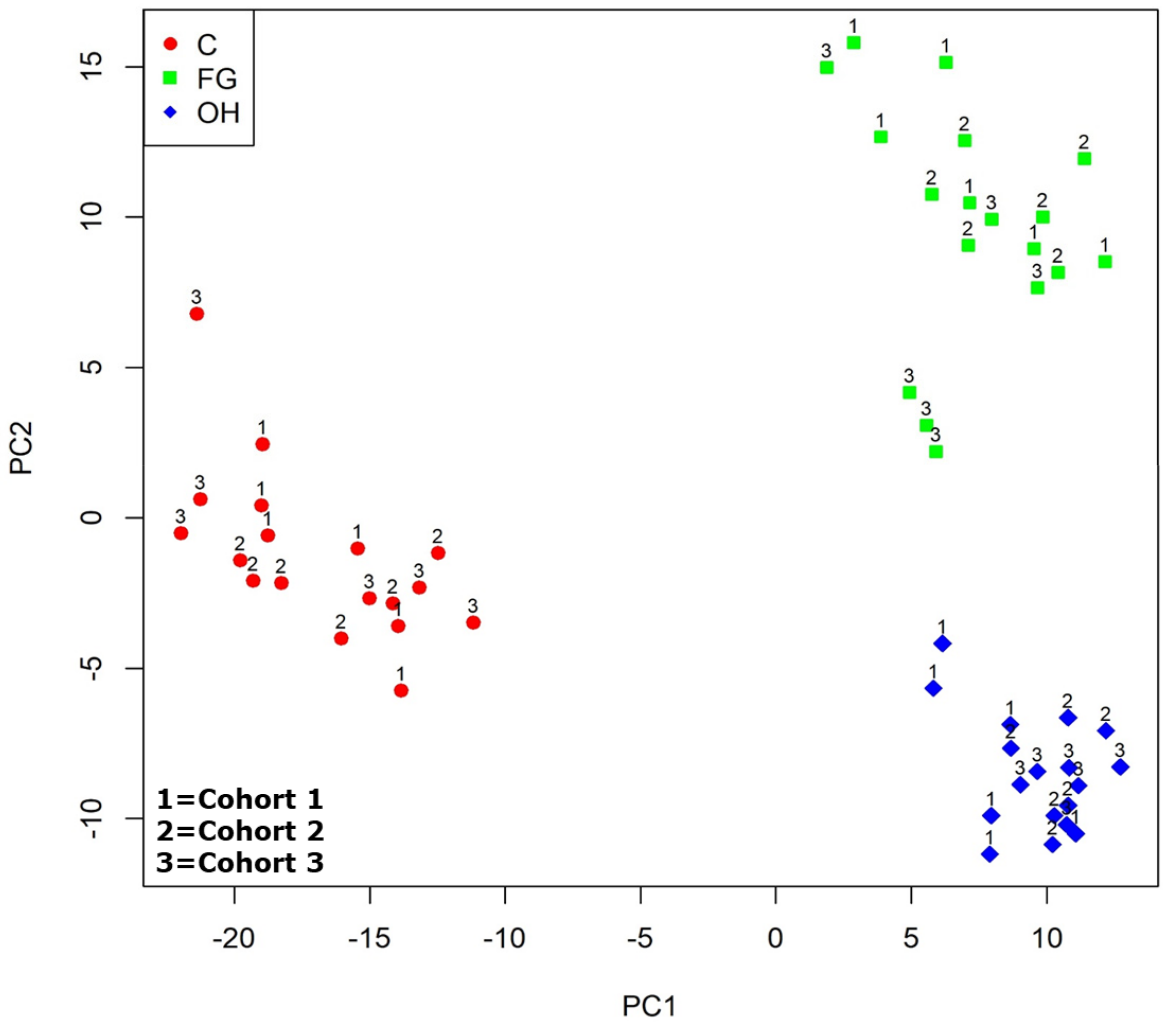
Principal component analysis of control, fireground, and overhaul gene expression data following surrogate variables removal. Principal components 1 and 2 are shown with control samples are represented by circles (red color). The fireground samples are represented by squares (lime color) and the overhaul samples represented as diamonds (blue color). The numeric labels 1, 2, and 3 indicate the cohort.

To determine the transcriptomic relationship between exposure/control groups, high-throughput sequencing was used to delineate global gene expression. Table 2 indicates the number and types of 41,786 gene entities in the genome and the 16,261 genes remaining after filtering. Principle Components Analysis clustering after removing the effects of the eight surrogate variables (Fig 1) shows significant separation between all three groups (C, FG, and OH) based on the distance between clusters plotted on PC1 and PC2.

**Table 2:** Number of different gene entities in NCBI Mus musculus GRCm38.p3 gene annotations.

Entity Type	Number in Genome	Number After Filtering
mRNA	21,198	13,743
ncRNA	12,285	1,342
Exon	4,008	144
Misc_RNA	1,988	940
Precursor_RNA	1,187	17
V_segment	535	49
tRNA	413	9
J_segment	94	1
rRNA	35	2
D_segment	23	0
C_region	20	13
Total	41,786	16,261

### Gene expression in lung after overhaul exposures is markedly different from fireground exposures

Table 3 shows the number of significant differentially expressed genes overall and broken down by each of the three fires. Overall, mice exposed to the overhaul environment resulted in a dramatically differential gene expression than mice kept at the fireground, modulating 3,852 genes. However, it is also apparent there was significant fire-to-fire variation in the gene expression, ranging from 3,460 on Fire 1 to 698 on Fire 3, although the majority of significantly changed genes on Fire 1 were trending the same direction on Fires 2 and 3, leading to overall FG vs. OH significance.

**Table 3:** Number of genes significantly up- or down- regulated (FDR p-value < 0.05) by overhaul (OH) exposure compared with fireground (FG).

Treatment	Up	Down	Total
FG vs OH	1,890	1,962	3,852
FG.1 vs OH.1	1,651	1,809	3,460
FG.2 vs OH.2	557	687	1,244
FG.3 vs OH.3	356	342	698

Direction of significantly expressed genes refers to expression level in OH compared to FG as the baseline.

Table 4 highlights these differentially expressed genes that display a greater than ± 50%-fold change (FC). This list consists of 43 up-regulated and 43 down-regulated genes. Importantly, the top 5 up-regulated genes link to cancer or immunomodulation, including calpain 11 (Capn11), immunoglobulin kappa chain variable 5–43 (Igkv5-43), immunoglobulin heavy constant alpha (Igha), immunoglobulin heavy variable 1–26 (Ighv1-26), and immunoglobulin heavy constant gamma 2B (Ighg2b) [29–33]. In correlate, several down-regulated genes are important to immune and cancer defense, specifically tumor necrosis factor (ligand) superfamily member 9 (Tnfsf9), tumor necrosis factor receptor superfamily member 13c (Tnfrsf13c), and S100 calcium binding protein A8 (S100a8) [34–36].

**Table 4:** List of significant differentially expressed genes for FG vs OH.

Gene Symbol	Entrez ID	Gene Name	Fold Change
Igha	238447	immunoglobulin heavy constant alpha	4.24
Igkv5-43	381783	immunoglobulin kappa chain variable 5–43	2.89
Ighv1-26	629884	immunoglobulin heavy variable 1–26	2.68
Capn11	268958	calpain 11	2.54
Ighg2b	16016	immunoglobulin heavy constant gamma 2B	2.53
Rorc	19885	RAR-related orphan receptor gamma	2.48
Gzmk	14945	granzyme K	2.34
Igj	16069	immunoglobulin joining chain	2.32
Ighg1	16017	immunoglobulin heavy constant gamma 1 (G1m marker)	2.09
Dnase2b	56629	deoxyribonuclease II beta	2.08
Rasd1	19416	RAS, dexamethasone-induced 1	1.99
Akr1c14	105387	aldo-keto reductase family 1, member C14	1.93
Igkv1-135	243420	immunoglobulin kappa variable 1–135	1.85
Cry1	12952	cryptochrome 1 (photolyase-like)	1.83
Gpr137c	70713	G protein-coupled receptor 137C	1.82
Igkv2-109	628268	immunoglobulin kappa variable 2–109	1.75
Gm11827	100503518	predicted gene 11827	1.75
Ighv3-6	780829	immunoglobulin heavy variable 3–6	1.75
Ctla4	12477	cytotoxic T-lymphocyte-associated protein 4	1.73
Doc2b	13447	double C2, beta	1.73
Ddit4	74747	DNA-damage-inducible transcript 4	1.73
Ces1g	12623	carboxylesterase 1G	1.72
Nav3	260315	neuron navigator 3	1.71
Ighg2c	404711	immunoglobulin heavy constant gamma 2C	1.69
Rasl10b	276952	RAS-like, family 10, member B	1.68
Ryr3	20192	ryanodine receptor 3	1.67
Gkn3	68888	gastrokine 3	1.66
Lonrf3	74365	LON peptidase N-terminal domain and ring finger 3	1.64
Csrnp1	215418	cysteine-serine-rich nuclear protein 1	1.63
Gzmb	14939	granzyme B	1.62
Tmem252	226040	transmembrane protein 252	1.61
Serpini1	20713	serine (or cysteine) peptidase inhibitor, clade I, member 1	1.59
Ighv9-3	780825	immunoglobulin heavy variable V9-3	1.58
Scn2b	72821	sodium channel, voltage-gated, type II, beta	1.58
Sox8	20681	SRY (sex determining region Y)-box 8	1.57
Gadd45g	23882	growth arrest and DNA-damage-inducible 45 gamma	1.56
BB123696	105404	expressed sequence BB123696	1.55
Adm	11535	adrenomedullin	1.54
Gal3st3	545276	galactose-3-O-sulfotransferase 3	1.53
Ajap1	230959	adherens junction associated protein 1	1.52
Abhd12b	100504285	abhydrolase domain containing 12B	1.52
Kcnip3	56461	Kv channel interacting protein 3, calsenilin	1.51
Cebpd	12609	CCAAT/enhancer binding protein (C/EBP), delta	1.50
Ly6c2	100041546	lymphocyte antigen 6 complex, locus C2	-1.51
Col5a3	53867	collagen, type V, alpha 3	-1.51
Slc22a3	20519	solute carrier family 22 (organic cation transporter), member 3	-1.51
Muc5b	74180	mucin 5, subtype B, tracheobronchial	-1.52
Mnd1	76915	meiotic nuclear divisions 1	-1.53
Tnfrsf13c	72049	tumor necrosis factor receptor superfamily, member 13c	-1.54
Artn	11876	artemin	-1.54
Ccno	218630	cyclin O	-1.55
Fbp2	14120	fructose bisphosphatase 2	-1.55
Olfm2	244723	olfactomedin 2	-1.55
Sp5	64406	trans-acting transcription factor 5	-1.56
Tnc	21923	tenascin C	-1.56
Loxl1	16949	lysyl oxidase-like 1	-1.56
Elovl1	54325	elongation of very long chain fatty acids (FEN1/Elo2, SUR4/Elo3, yeast)-like 1	-1.56
AI854703	243373	expressed sequence AI854703	-1.56
Retnla	57262	resistin like alpha	-1.56
Muc20	224116	mucin 20	-1.56
Cftr	12638	cystic fibrosis transmembrane conductance regulator	-1.57
Tubb4a	22153	tubulin, beta 4A class IVA	-1.58
Clmp	71566	CXADR-like membrane protein	-1.58
Ccna1	12427	cyclin A1	-1.58
Mfsd4	213006	major facilitator superfamily domain containing 4A	-1.59
Tmem132c	208213	transmembrane protein 132C	-1.59
Cpm	70574	carboxypeptidase M	-1.59
Tulp1	22157	tubby like protein 1	-1.59
Ppp1r3c	53412	protein phosphatase 1, regulatory (inhibitor) subunit 3C	-1.60
Tnfsf9	21950	tumor necrosis factor (ligand) superfamily, member 9	-1.61
Aoc1	76507	amine oxidase, copper-containing 1	-1.61
Ccdc42	276920	coiled-coil domain containing 42	-1.64
Nxph3	104079	neurexophilin 3	-1.64
Unc80	329178	unc-80, NALCN activator	-1.65
Scn3a	20269	sodium channel, voltage-gated, type III, alpha	-1.65
Klk14	317653	kallikrein related-peptidase 14	-1.66
Mcidas	622408	multiciliate differentiation and DNA synthesis associated cell cycle protein	-1.66
Mmp9	17395	matrix metallopeptidase 9	-1.67
Olfr1342	258708	olfactory receptor 1342	-1.67
Camkk1	55984	calcium/calmodulin-dependent protein kinase kinase 1, alpha	-1.67
Gmnc	239789	geminin coiled-coil domain containing	-1.67
S100a8	20201	S100 calcium binding protein A8 (calgranulin A)	-1.69
Ppp1r1b	19049	protein phosphatase 1, regulatory (inhibitor) subunit 1B	-1.71
Pbld1	68371	phenazine biosynthesis-like protein domain containing 1	-1.72
Slc26a4	23985	solute carrier family 26, member 4	-1.73
Rasd2	75141	RASD family, member 2	-1.75

### Heatmap display shows global gene expression differences in lung tissue exposed to the overhaul environment

**Fig 2:**
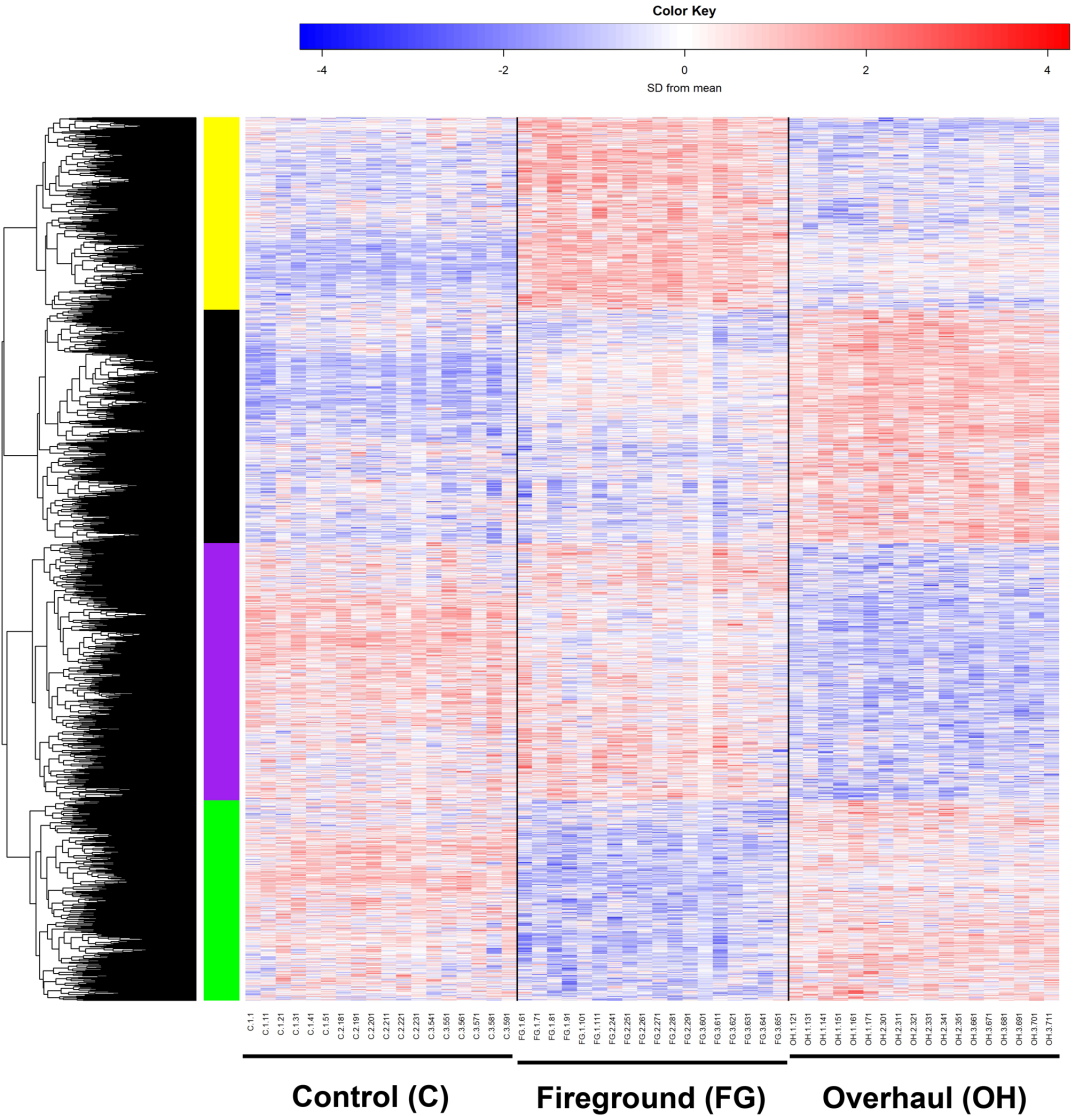
Heatmap showing changes in global gene expression. The overall expression patterns across all three treatments groups were visualized for the 3,852 genes with OH vs. FG FDR p-value < 0.05 using a heatmap. Each row represents one gene and each column is one individual mouse, grouped by treatment. The color scale represents standard deviations from the mean expression level across all samples with greater expression represented in red and lesser expression by blue relative to the mean.

**Fig 3:**
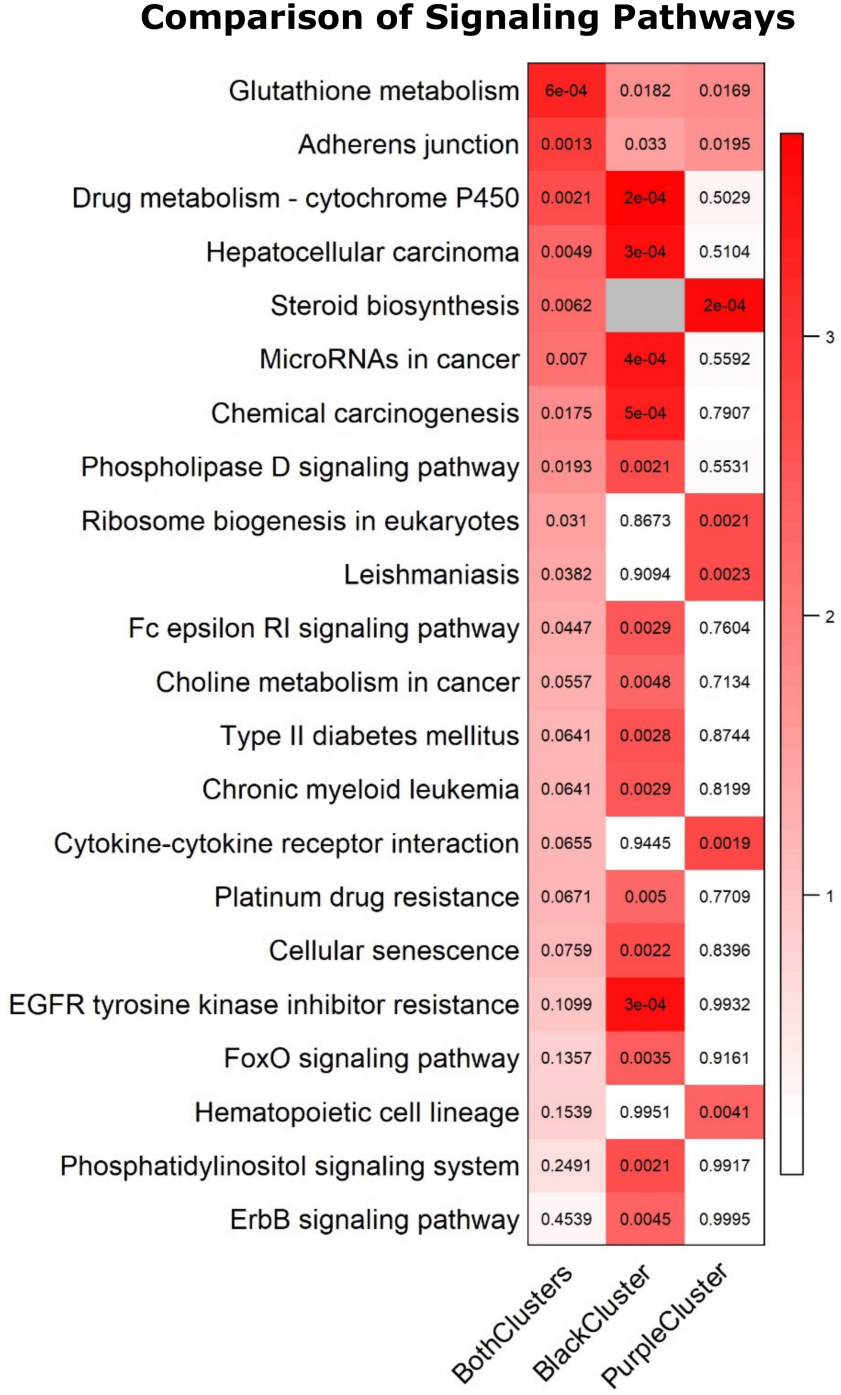
Significant over-represented KEGG pathways amongst overhaul treated samples. Over-representation testing completed based on purple and black gene set clusters, as well as both clusters combined, identified from heatmap (Fig 2) using Kyoto Encyclopedia of Genes and Genomes (KEGG) pathways. Twenty-two pathways had more significant genes than expected by chance in at least one of the three comparisons (raw p-value < 0.005). The color of the box represents the –log10(p-value) to give more significant values darker color while the actual p-values are printed inside each box; the grey box indicates no genes in the black cluster mapped to that pathway.

The 3,852 significantly expressed genes in the group exposed to the overhaul environment vs fireground (Table 3) were visualized in a heatmap to see the expression patterns across all three groups. Two main heatmap patterns were apparent based on differences in exposure (Fig 2). Pattern 1 shows marked similarities in the C and FG groups when compared to the OH group for the genes located in the purple and black bars (n genes = 1,122 and 1,017, respectively). Pattern 2 shows marked similarities in the C and OH groups compared to the FG group for genes located in the yellow and green bars (n genes = 839 and 874, respectively). Finally, KEGG (Kyoto Encyclopedia of Genes and Genomes) analysis (Fig 3) of the black, purple, and both (black and purple combined) gene clusters from Fig 2 shows 22 significantly over-represented cellular pathways [37]. The black cluster contained 68% (15/22) of the over-represented pathways while the purple cluster contained 23% (5/22) with 9% (2/22) overlapping between both clusters.

## Discussion

Fire suppression is associated with high rates of duty-related sudden cardiac death [38,39]. In addition, firefighters are at increased risk for developing lung disease [40,41]and cancer [2,3,42]. While the etiologies of lung disease and cancer are thought to be linked to toxicant exposure during fire suppression and overhaul activities [16,43], mechanistically little is known about why firefighters show these increased incidences or what aspects of firefighting exacerbates disease risks. Using a mouse model of exposure sans airway protection, the impact of environmental exposure during overhaul on lung gene expression was assessed to better define pathways that are potentially critical to firefighter-related chronic illnesses. Our major finding is that working in an overhaul environment without breathing protection is associated with changes in transcripts with links to respiratory diseases including asthma, COPD and cancer. Importantly, these changes occurred in the absence of obvious increases in the poisonous gases HCN and H_2_S.

As expected, mice absent from the fireground (C group) or on the fireground but well distanced from the overhaul activities (FG group) showed greater similarity in gene expression than mice exposed to the overhaul environment (OH group). Interestingly, the C vs. FG group comparison showed more gene expression dissimilarity than anticipated (Fig 2). While transportation stress in mice is a well-described phenomenon [44,45], the magnitude of this effect from a gene transcription perspective is not currently known, but appears to need further study. In addition to transportation stress, the FG group was also exposed to fire apparatus lights, fireground sounds, and, potentially, light smoke. All or anyone of these could be a potential confound.

Gene listed in Table 4 are associated with immune and inflammatory pathways differentially expressed in the OH vs FG group. Interestingly, 50% of these genes were downregulated. An expected upregulation of pro-inflammatory genes, [46] downstream of NF-κB, was not observed, which was unexpected. In fact, the two principal cytokine mediators of innate immunity [47], namely IL-1βnot shown) and TNF were down-regulated. In contrast, a small group of cancer-associated genes were up-regulated during overhaul including: Calpain 11 (Capn11), RAR-Related Orphan Receptor Gamma (Rorc), and Deoxyribonuclease II Beta (Dnase2b) [29,48,49]. This pattern of gene expression accounts for the overrepresentation of pathways linking immune dysregulation to cancer (Fig 3), and suggests that working in the overhaul environment without airway protection, even when visibly “clear”, poses a danger to lung health. These findings may also add insight into the increased incidence of respiratory diseases and cancer that is reported in the fire service [50,51].

Other than CO, gases were measured at levels below the recommended limits for an 8–10 hr occupational exposure even when peak values were factored. CO, however, exceeded the NIOSH REL TWA (10 hour), and CO peak levels were above OSHA PEL TWA (8 hour). Since CO never approached the NIOSH ceiling value of 200 ppm and peak values were well below the IDLH of 1200 ppm [52,53], many fire services would clear firefighters for SCBA face piece removal in the overhaul conditions experienced by mice in this study. Additionally, for fire departments without quantitative requirements, the qualitative observation that the area was visibly clear of smoke would allow for unmasking.

Given that CO levels did not appear to correlate with the differential gene expression observed, several other well-described fireground contaminants could be responsible for the results including benzene and polycyclic aromatic hydrocarbons [54]. Unfortunately, portable monitoring for said toxicants was not available to this study. In sum, changes in lung gene expression appear relatively substantial in the unprotected mouse during overhaul. Further investigation is warranted to better understand how activation of potentially deleterious pathways in the mouse lung translate to pulmonary diseases in individuals exposed to the fireground.

## Supporting information

S1 Checklist (PDF)
